# Metabolic Engineering *Camelina sativa* with Fish Oil-Like Levels of DHA

**DOI:** 10.1371/journal.pone.0085061

**Published:** 2014-01-21

**Authors:** James R. Petrie, Pushkar Shrestha, Srinivas Belide, Yoko Kennedy, Geraldine Lester, Qing Liu, Uday K. Divi, Roger J. Mulder, Maged P. Mansour, Peter D. Nichols, Surinder P. Singh

**Affiliations:** 1 CSIRO Food Futures National Research Flagship, Canberra, Australian Capital Territory, Australia; 2 CSIRO Food Futures National Research Flagship, Hobart, Tasmania, Australia; 3 CSIRO Materials Science and Engineering, Clayton, Victoria, Australia; University of Texas, United States of America

## Abstract

**Background:**

Omega-3 long-chain (≥C20) polyunsaturated fatty acids (ω3 LC-PUFA) such as eicosapentaenoic acid (EPA) and docosapentaenoic acid (DHA) are critical for human health and development. Numerous studies have indicated that deficiencies in these fatty acids can increase the risk or severity of cardiovascular, inflammatory and other diseases or disorders. EPA and DHA are predominantly sourced from marine fish although the primary producers are microalgae. Much work has been done to engineer a sustainable land-based source of EPA and DHA to reduce pressure on fish stocks in meeting future demand, with previous studies describing the production of fish oil-like levels of DHA in the model plant species, *Arabidopsis thaliana*.

**Principal Findings:**

In this study we describe the production of fish oil-like levels (>12%) of DHA in the oilseed crop species *Camelina sativa* achieving a high ω3/ω6 ratio. The construct previously transformed in Arabidopsis as well as two modified construct versions designed to increase DHA production were used. DHA was found to be stable to at least the T_5_ generation and the EPA and DHA were found to be predominantly at the *sn*-1,3 positions of triacylglycerols. Transgenic and parental lines did not have different germination or seedling establishment rates.

**Conclusions:**

DHA can be produced at fish oil-like levels in industrially-relevant oilseed crop species using multi-gene construct designs which are stable over multiple generations. This study has implications for the future of sustainable EPA and DHA production from land-based sources.

## Introduction

The omega-3 long-chain (≥C20) polyunsaturated fatty acids (ω3 LC-PUFA) EPA (eicosapentaenoic acid, 20∶5ω3) and DHA (docosahexaenoic acid, 22∶6ω3) are recognized for their strong health benefits. Developing an oilseed source of these fatty acids is desirable since these provide far stronger health benefits than the shorter chain terrestrial plant precursors of these fatty acids, α-linolenic acid (ALA, 18∶3ω3) and stearidonic acid (SDA, 18∶4ω3) [1]. Moreover, the conversion of these precursor fatty acids to long-chain variants occurs at surprisingly low levels in humans [2]. Importantly, studies have also found that high concentrations of ω6 fatty acids such as LA (linoleic acid, 18∶2ω6), GLA (γ-linolenic acid, 18∶3ω6) and ARA (arachidonic acid, 20∶4ω6) can decrease the bioconversion efficiency of C18 ω3 fatty acids to long-chain PUFA [2]. The production of ω3 LC-PUFA in land plants as been a long-standing goal of bioengineers. The first demonstrations of LC-PUFA production were published in 2004 and showed EPA and ARA production in leaf [3] and seed [4]. The first demonstrations of DHA production in seed were published soon after [5], [6]. Subsequent work resulted in increasing levels of production, particularly for EPA [7]–[9]. The first report of production of fish oil-like levels of DHA in seed was published in 2012 [10]. Progress has been extensively reviewed in recent years [11], [12].

**Figure 1:**
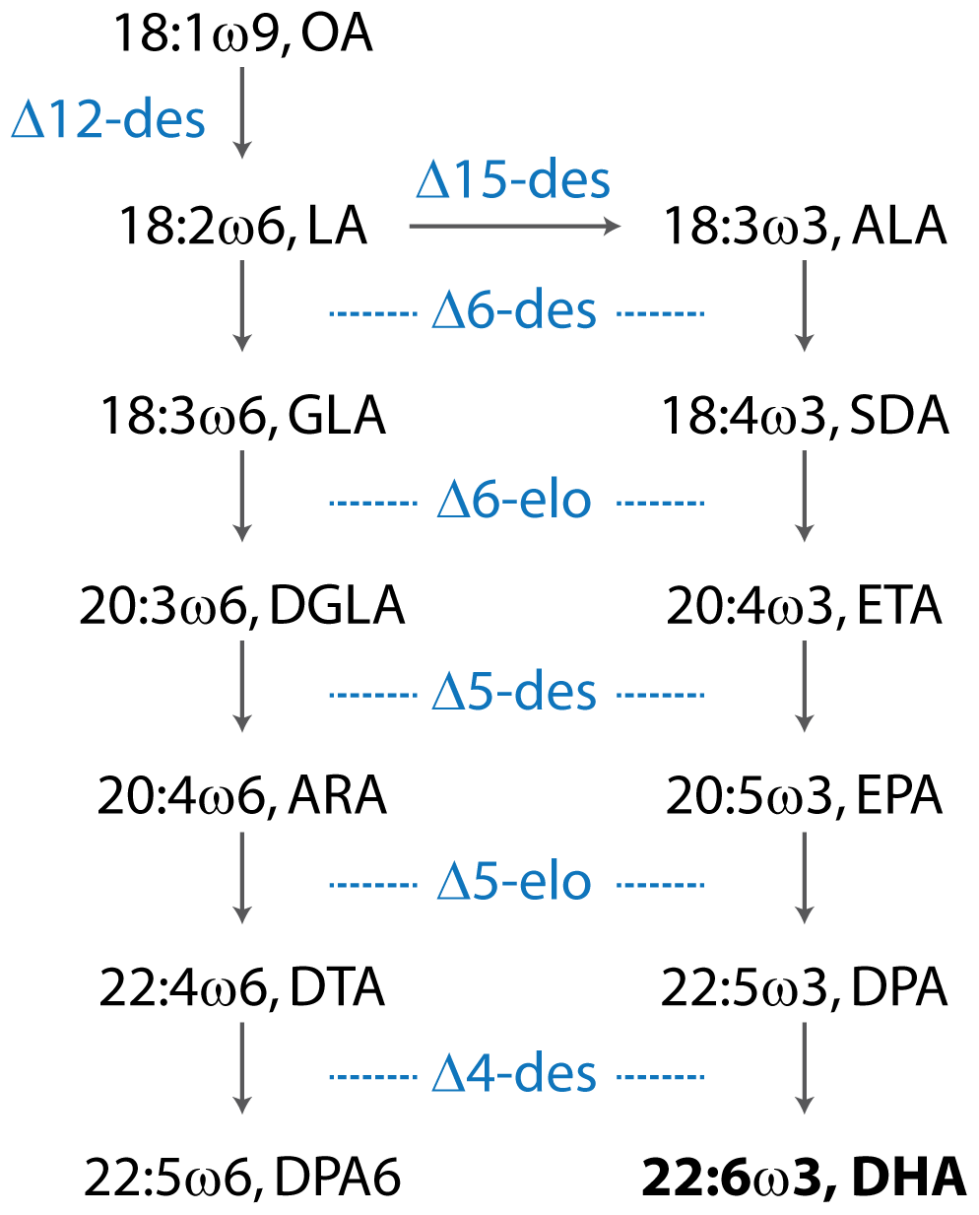
The parallel ω6 and ω3 Δ6-desaturase pathway for DHA synthesis described in this article. ‘Des’ denotes desaturase and ‘elo’ denotes elongase. OA, oleic acid; LA, linoleic acid; GLA, γ-linolenic acid; DGLA, dihomo-γ-linolenic acid; ARA, arachidonic acid; DTA, docosatetraenoic acid; DPA6, ω6 docosapentaenoic acid; ALA, α-linolenic acid; SDA, stearidonic acid; ETA, eicosatetraenoic acid; EPA, eicosapentaenoic acid; DPA, ω3 docosapentaenoic acid.

*Camelina sativa*, also known as Gold of Pleasure and False Flax, is an ancient cultivated oilseed crop [13], [14] with naturally high levels of the C18 ω3 ALA in seed oil. Commercial cultivation of the crop slowed significantly with the introduction of oilseed rape but has recently garnered significant attention as an underutilised species. Some *C. sativa* varieties have high oil content (in excess of 40%) and have strong agronomic performance on marginal lands [15] and approvals have recently been granted for *C. sativa* meal or oil use in food and animal feed applications in USA and Canada. In this article we describe the production of fish oil-like levels of DHA (12%) in *C. sativa* by the introduction of a transgenic Δ6-desaturase pathway (Figure 1) consisting of both yeast and microalgal genes to convert native OA (oleic acid, 18∶ω9), LA and ALA substrates to the beneficial ω3 LC-PUFA EPA and DHA.

## Materials and Methods

### Genes and expression vectors

**Figure 2:**
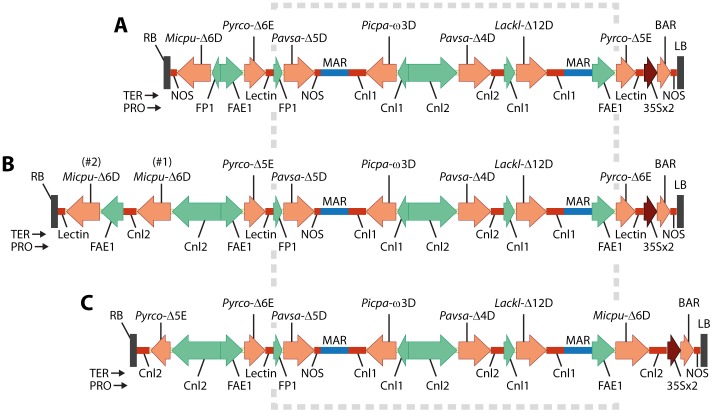
DHA pathway construct maps described in this study: A) GA7 parental construct; B) mod-F variant with an additional Δ6-desaturase, elongase coding regions switched and the original FP1 promoter replaced by Cnl2; C) mod-G with the Δ5-elongase and Δ6-desaturase coding regions switched. The box denotes the unchanged region. Abbreviations are: TER, terminator or polyadenylation region; PRO, promoter; NOS, *Agrobacterium tumefaciens* nopaline synthase terminator; FP1, *Brassica napus* truncated napin promoter; FAE1, *Arabidopsis thaliana* FAE1 promoter; Lectin, *Glycine max* lectin terminator; Cnl1 and Cnl2 denotes the *Linum usitatissimum* conlinin1 or conlinin2 promoter or terminator. MAR denotes the Rb7 matrix attachment region from *Nicotiana tabacum* and gene identities are given in the text and the Materials and Methods section.

Constructs mod-F and mod-G were made using a combination of DNA synthesis and restriction enzyme-based cloning starting with the previously described GA7 parent vector [10], Figure 2. Mod-F was made by replacing the *Sbf*I-flanked Δ5-elongase expression cassette near the left border with a Δ6-elongase expression cassette. The original Δ6-desaturase expression cassette and the adjacent Δ6-elongase promoter and coding region were then excised with *Asc*I + *Pme*I and replaced with the Cnl2:: Δ6-desaturase::NOS expression cassette plus the replacement Δ5-elongase promoter and coding region. The second Δ6-desaturase expression cassette was then added at the *Pme*I site as a *Pme*I-*Swa*I blunt-ended fragment to generate mod-F. Construct mod-G was generated by first replacing the original Δ6-desaturase expression cassette and the adjacent Δ6-elongase promoter and coding region (flanked by *Asc*I + *Pme*I) with the new Δ5-elongase expression cassette and adjacent Δ6-elongase coding region. The original Δ5-elongase expression cassette adjacent to the left border (flanked by *Sbf*I) was then replaced with the Δ6-desaturase expression cassette.

### Plant transformation

Constructs were transformed in *Agrobacterium tumefaciens* strain AGL1 and cultured at 28°C on a rotary shaker to appropriate growth phase. *C. sativa* (“Celine”) was transformed by a floral dip method adapted from Liu et al. [16]. Briefly, the freshly opened flower buds were dipped in *A. tumefaciens* solution for 15 s, wrapped in plastic film and left overnight in the dark at 24°C after which the plastic was removed.

### Lipid fractionation and fatty acid profile analysis

Total lipid was extracted from seeds using chloroform: methanol: 0.1 M KCl (2∶1∶1 v/v/v as described in [17]. Neutral lipid classes and polar lipid were fractionated from the total lipid on a TLC plate (Silica gel 60, MERCK) using a mixture of hexane: diethylether: acetic acid (70∶30∶1, v/v). The lipid bands were visualized under UV after spraying the plate with 0.001% primuline dissolved in acetone:water (80∶20 v/v). The individual lipid bands were identified on the basis of authentic lipid standards, which were run parallel to the seed lipid samples in the TLC, collected into separate glass vials and their fatty acid methyl esters (FAME) were prepared together with known amount of heptadecanoic acid as internal standard. FAMEs were analysed by GC as previously described [10] and individual lipids were quantified on the basis of the amounts of FAMEs produced from the known amount of internal standards used.

DHA levels in T_5_ seeds from 15 randomly selected homozygous plants grown in three glasshouses were analysed by One Way Analysis of Variance (ANOVA) using Sigmaplot (v12) by the Holm-Sidak method [18].

### Expression analysis

RNA was extracted from developing seed taken from three plants of a high DHA line transformed with either GA7, mod-F or mod-G using RNeasy Plant Mini kit (Qiagen, Hilden, Germany). Total RNA (1 µg) was converted to cDNA using First-Strand cDNA synthesis mix (OriGene Inc., Australia). Diluted cDNA (0.2×) was used for quantitative real-time PCR by Bio-Rad CFX Real-Time System (BIO-RAD, USA) using iQSYBR Green Supermix (BIO-RAD). Reactions were carried with initial denaturation at 95°C for 3 min, followed by 35 cycles of 95°C for 10 sec, 58°C for 30 sec and 68°C for 30 sec. Target gene expression was normalized to the endogenous *C. sativa* HMG gene in the Bio-Rad CFX Manager software.

### ^13^C NMR analysis

A hexane-extracted oil (>99% TAG, confirmed by TLC-FID; results not shown) was obtained by sequential extraction of crushed seeds and prior to analysis was stored at -18°C. Samples were warmed to room temperature 1 h prior to NMR sample preparation. 100 mg oil was dissolved in deuteriochloroform containing 25 mM Tris(acetylacetonate) chromium(III) as a relaxation agent (0.6 mL). The solutions were transferred to 5 mm O.D. NMR tubes (New Era NE-UL5-7) and sealed with PTFE lids. Solutions for NMR spectroscopy were stored at 4°C until they were inserted into the magnet. Quantitative ^13^C NMR spectra were acquired on a Bruker BioSpin Av500 NMR spectrometer equipped with a 5 mm ^1^H-^13^C/^15^N triple-resonance inverse probe operating at 125.8 MHz for ^13^C. The data were acquired and processed in Bruker BioSpin TopSpin v3.2. The samples were maintained at 25°C during acquisition. 128 k data points were collected over a spectral width of 26.3 kHz summed over 46 k scans. Inverse-gated, bilevel adiabatic ^1^H-decoupling was employed with an acquisition time of 2.49 s and a recycle delay of 2.5 s. Data were processed to 128 k data points using a Gaussian multiplication with a Gaussian position factor of 0.12 and a line broadening of −0.15 Hz prior to Fourier transformation; a 5th-order polynomial baseline correction was applied to each spectrum. Spectra were referenced to the peak arising from C1 of 22∶6ω3 in the *sn*-2 position of TAG at 172.13 ppm [19] and the signals assigned using the published assignments [20]. The raw data were processed in a similar fashion in triplicate and the mean and standard deviations calculated.

## Results and Discussion

### Construct design and manufacture

Use of construct pJP3416_GA7 (GA7) to generate DHA-containing seeds in *A. thaliana* has previously been described [10]. Two GA7 construct variants, referred to here as mod-F and mod-G, were designed to improve the efficiency of the Δ6-desaturase and Δ6-elongase steps. The core GA7 sequence was left intact (Figure 2a) with only the terminal regions that contained the genes of interest modified. Specifically, the GA7 terminal FP1/NOS promoter/terminator pair was replaced with the *Linum usitatissimum* conlinin2 (Cnl2) promoter/terminator pair in both new variants. The mod-F changes consisted of the switching of the two elongase coding regions as well as the addition of a second *Micromonas pusilla* Δ6-desaturase coding region with different codon usage to the original GA7 version (Figure 2b). In addition to the conlinin2 cassette changes described above, the Δ6-desaturase and Δ5-elongase coding regions were also switched in mod-G (Figure 2c).

Conversion of ALA to SDA (Δ6-desaturation) had previously been identified as a bottleneck in DHA production in *A. thaliana* and in this study we tested both the effect of using different promoters (*A. thaliana* FAE1 and *L. usitatissimum* Cnl2) and the addition of a second expression cassette with different gene codon usage to avoid gene silencing. The changes made in mod-G also tested whether the expression cassette adjacent the right border was intrinsically compromised [21] by switching the Δ6-desaturation and Δ5-elongase cassettes. Similarly, the Δ5-elongase had previously been shown to have very high activity whilst the Δ6-elongase had lower activity. The changes in mod-F included switching the two elongase coding regions to test whether this was due to the expression cassette rather than the gene.

### Camelina sativa transformation

Constructs were sequence confirmed and transformed in *Agrobacterium* strain AGL1 before floral dip of *C. sativa*. After the floral dip, plants were grown to maturity, seed harvested, and germinated in soil trays. Established seedlings (7–10 days) were sprayed with 0.1% BASTA herbicide (250 g/L glufosinate ammonium; Bayer Crop Science Pty Ltd, VIC Australia) to kill plants not expressing the selectable marker gene. The original GA7 construct was used to establish the *C. sativa* transformation protocol in the lab and was transformed before the mod-F and mod-G constructs.

### Transgenic *C. sativa* can accumulate 12% DHA in seed oil

**Figure 3:**
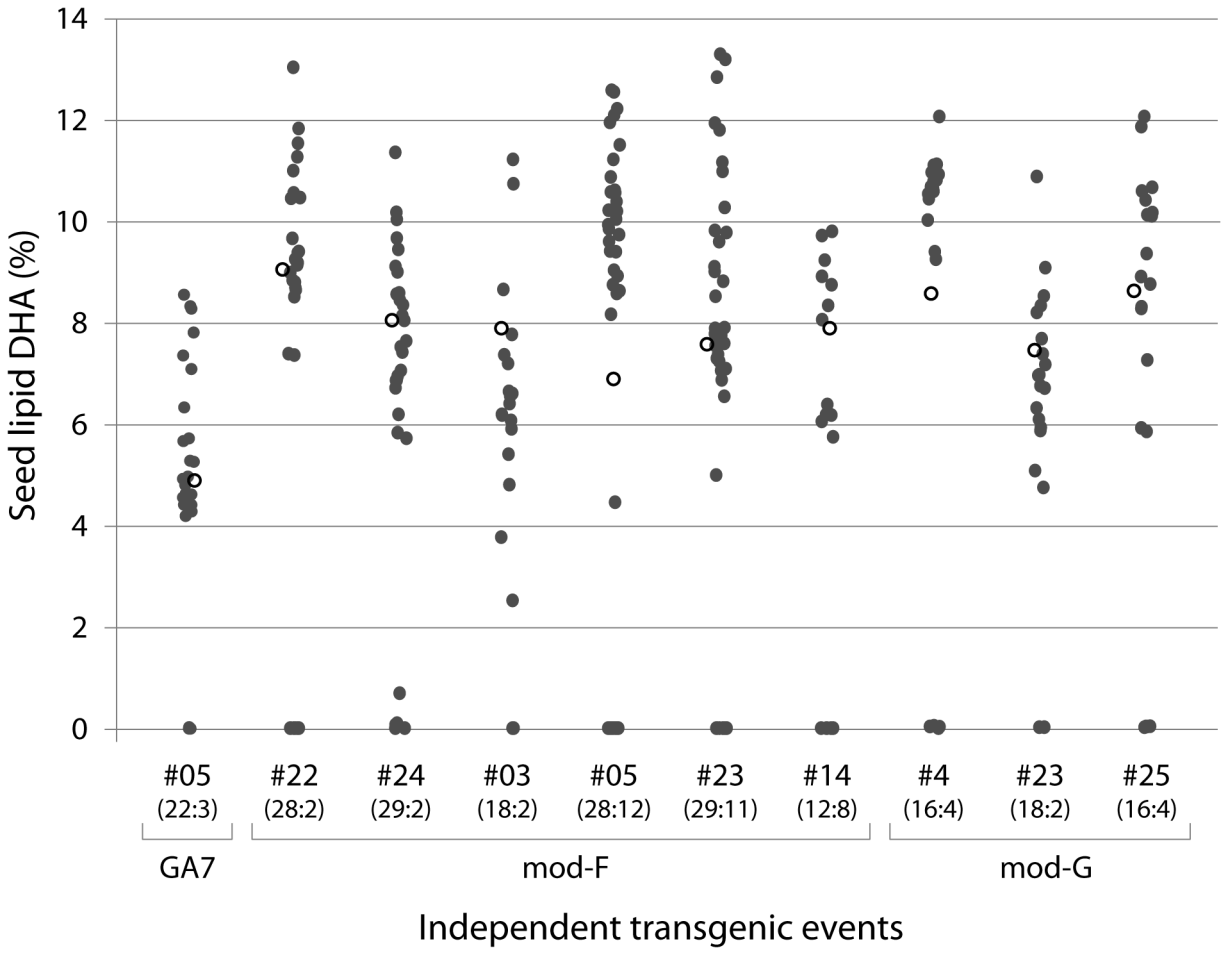
Relative level of DHA (as % of total fatty acids) in single seeds from independent T_2_ *Camelina sativa* events. The transgenic to null ratio is shown in parenthesis under the event number (note that not all zero DHA seeds were null). Open circles denote the DHA level in a pooled batch of 20 seeds (analysed separately).

BASTA-resistant seedlings were grown to maturity before seed was harvested and analysed for fatty acid profile. Analysis of the fatty acid profile was also performed on single seeds from selected (higher pooled DHA) lines to rapidly get an indication of both maximum DHA production and locus number based on the segregation ratio of DHA-producing transgenic seeds to null seeds (Figure 3). The DHA level in single seeds from several independent events exceeded 12%. The transgenic:null ratio of these lines was found to be between approximately 3∶1 and 15∶1. Analysis of representative fatty acid profiles from the top DHA samples from each construct (Table 1) found only 1.2–1.4% GLA with no other new ω6 PUFA detected. In contrast, new ω3 PUFA (SDA) and new ω3 LC-PUFA (ETA, EPA, DPA, DHA) were found to accumulate to 18.5% (GA7), 25.8% (mod-F) and 21.9% (mod-G). The DHA levels in these lines were 9.6%, 12.4% and 11.5%, respectively.

**Table 1:** Representative fatty acid profiles of seed lipids from independent transgenic parental, GA7, mod-F and mod-G lines (T_2_ seeds with the highest DHA levels).

	Parental	GA7	Mod-F	Mod-G
16∶0	8.0±0.2	8.9±0.3	8.2±0.1	9.5±0.6
18∶0	3.5±0.2	4.1±1.1	3.5±0.0	4.0±0.1
18∶1ω7	1.4±0.1	1.7±0.1	1.2±0.1	1.2±0.1
20∶0	2.4±0.4	1.4±0.5	1.0±0.1	2.1±0.2
20∶1ω9/11	10.2±0.5	7.6±0.6	8.1±0.2	7.3±0.9
Minor	5.6	3.1	2.3	4.3
OA	9.8±0.2	6.3±0.2	9.5±0.2	4.4±0.3
LA	18.1±0.6	6.7±0.1	8.4±0.3	11.5±1.3
ALA	38.2±1.4	39.5±2.0	29.4±0.5	29.6±3.8
**Omega-6**				
GLA	–	–	1.2±0.1	1.4±0.1
20∶2ω6	1.5±0.0	0.7±0.0	0.6±0.0	1.3±0.1
DGLA	–	–	–	–
ARA	–	–	–	–
22∶4ω6	–	–	–	–
DPA6	–	–	–	–
**Omega-3**				
SDA	–	7.4±0.4	8.9±0.5	8.5±1.8
20∶3ω3	1.3±0.1	1.5±0.2	0.8±0.0	1.5±0.2
ETA	–	0.3±0.1	0.4±0.1	0.4±0.2
EPA	–	0.8±0.4	3.3±0.1	0.8±0.4
DPA	–	0.4±0.0	0.8±0.1	0.7±0.4
**DHA**	–	**9.6**±**0.2**	**12.4**±**0.4**	**11.5**±**0.8**
Δ**12-des**	86%	91%	87%	94%
Δ**15-des**	69%	90%	87%	83%
**ω3** Δ**6-des**	–	32%	47%	43%
**ω3** Δ**6-elo**	–	60%	66%	61%
**ω3** Δ**5-des**	–	97%	98%	97%
**ω3** Δ**5-elo**	–	93%	80%	94%
**ω3** Δ**4-des**	–	96%	94%	94%

The errors denote standard deviation of triplicate samples. Apparent conversion efficiencies shown at the bottom describe the ω3 pathway and are calculated as the sum of product FAs/sum of substrate + product FAs.

Δ6-desaturation was found to be lower in the GA7 lines than the mod-F and mod-G lines (32% vs 47% and 43%) and this resulted in a reduction of ALA in the mod-F and mod-G lines relative to GA7. Another noteworthy difference was the accumulation of EPA in the mod-F seed (3.3% vs 0.8% in the other two transgenic lines) and this was reflected in the reduced Δ5-elongation observed in mod-F (80%) relative to the other lines (93% and 94%). There was a slight increase in Δ6-elongation in these lines (66% vs 60% and 61%) although the amount of SDA actually increased due to the slightly more active Δ6-desaturation.The distribution of DHA between the seed lipid fractions was also examined (Table 2). Polar lipids were found to comprise 3.0% of the total seed lipids and contained 3.7% DHA.

**Table 2:** Representative fatty acid profiles of triacylglycerol (TAG), polar lipids, free fatty acids (FFA) and diacylglycerol (DAG) in T_5_ GA7 seed.

	TAG (96.3%)	Polar Lipids (3.0%)	FFA (0.3%)	DAG (0.4%)
16:0	7.1	26.7	24.5	19.3
18:0	4.2	8.0	23.4	12.6
18:1ω7	1.1	2.6	1.0	1.3
20:0	1.9	0.5	6.7	4.9
20:1ω9/11	8.6	1.1	2.1	8.6
Minor	2.9	2.4	5.3	5.2
				
OA	6.7	0.9	5.1	7.1
LA	6.7	2.1	4.8	5.3
ALA	41.2	42.3	20.8	22.5
				
**Omega-6**				
GLA	0.2	0.1	0.1	0.1
20:2ω6	0.7	0.4	0.4	0.7
DGLA	-	-	-	-
ARA	-	-	-	-
22:4ω6	-	-	-	-
DPA6	-	-	-	-
				
**Omega-3**				
SDA	6.4	5.2	2.1	4.0
20:3ω3	1.4	1.6	0.6	1.0
ETA	0.4	0.3	0.2	0.3
EPA	0.6	0.2	0.1	1.1
DPA	0.2	1.9	0.3	0.3
**DHA**	**9.7**	**3.7**	**2.5**	**5.7**

The relative quantity of each fraction as a percentage of total seed lipids is shown in parentheses).

**Figure 4:**
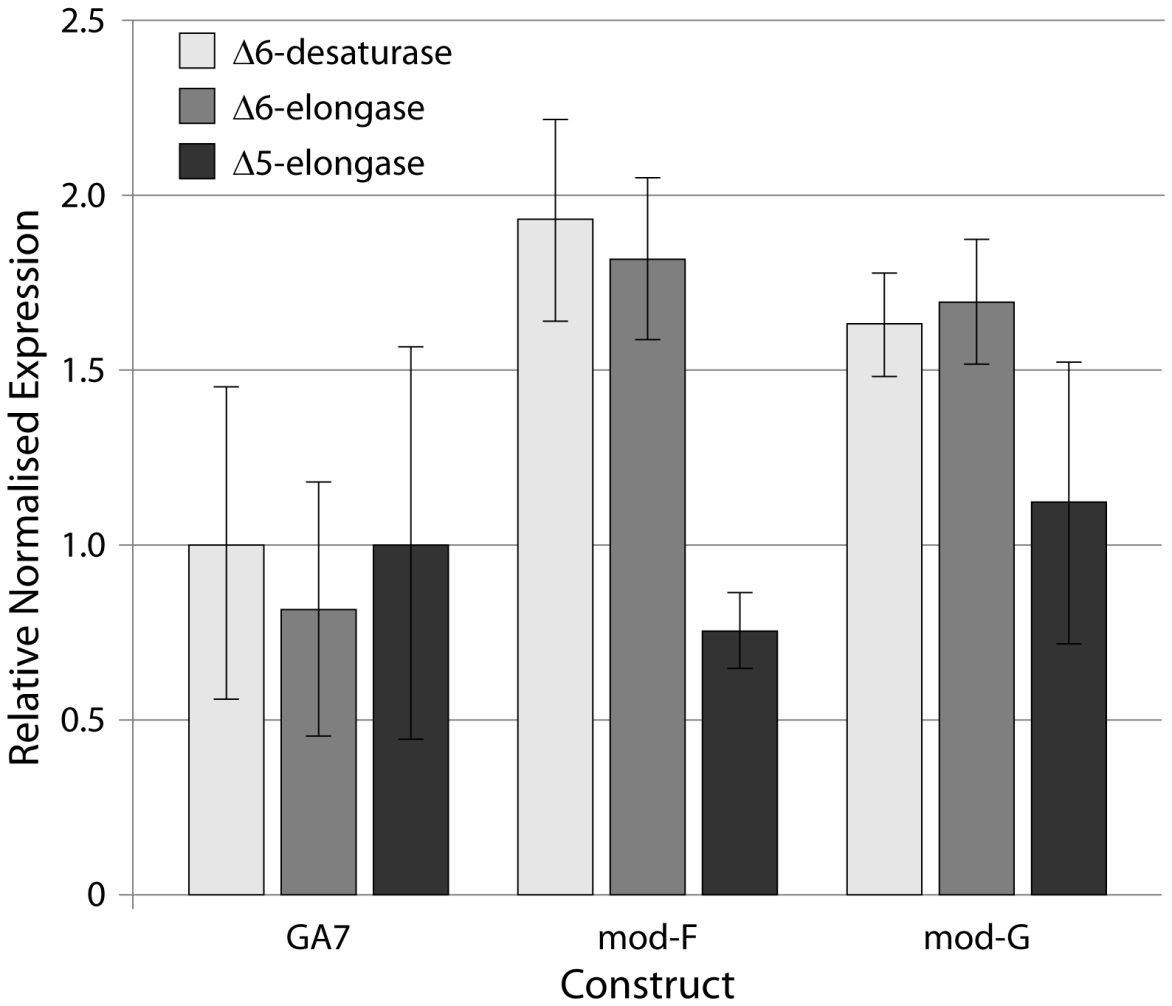
Average relative expression levels of the transgenic Δ6-desaturase, Δ6-elongase and Δ5-elongase from three biological replicates of transgenic *C. sativa* events transformed with constructs GA7, mod-F or mod-G. Error bars represent standard error. The data shown is for the Δ6-desaturase #1 for the mod-F construct (Figure 2) with the #2 desaturase found to have expression levels similar to the #1 gene across multiple events.

Whilst the focus of this study was the demonstration of DHA production in an oilseed crop species, the differences in gene activity noted above were also interesting from a construct design perspective. First, switching the Δ6- and Δ5-elongase coding region locations in mod-F resulted in the desired profile change with more EPA accumulated due to lower Δ5-elongation. A concomitant increase in Δ6-elongation was observed but this did not result in lower SDA levels. This was due to an increase in Δ6-desaturation in mod-F caused by adding an extra *M. pusilla* Δ6-desaturase expression cassette as well as by replacing the truncated napin promoter (FP1) with a more highly active *L. usitatissimum* Cnl2 promoter. The relatively moderate increase in Δ6-desaturation observed in mod-G was caused by capitalising on the highly expressed Δ5-elongase cassette in GA7. Switching the positions of the Δ6-desaturase and Δ5-elongase coding regions resulted in greater Δ6-desaturation. Δ5-elongase was not reduced in this instance due to the replacement of the FP1 promoter with the Cnl2 promoter. These functional changes were reflected in changes in the relative expression levels of the Δ6-desaturase, Δ6-elongase and Δ5-elongase genes in developing seeds from the three sample sets (Figure 4).

### The DHA trait is stable over multiple generations

**Figure 5:**
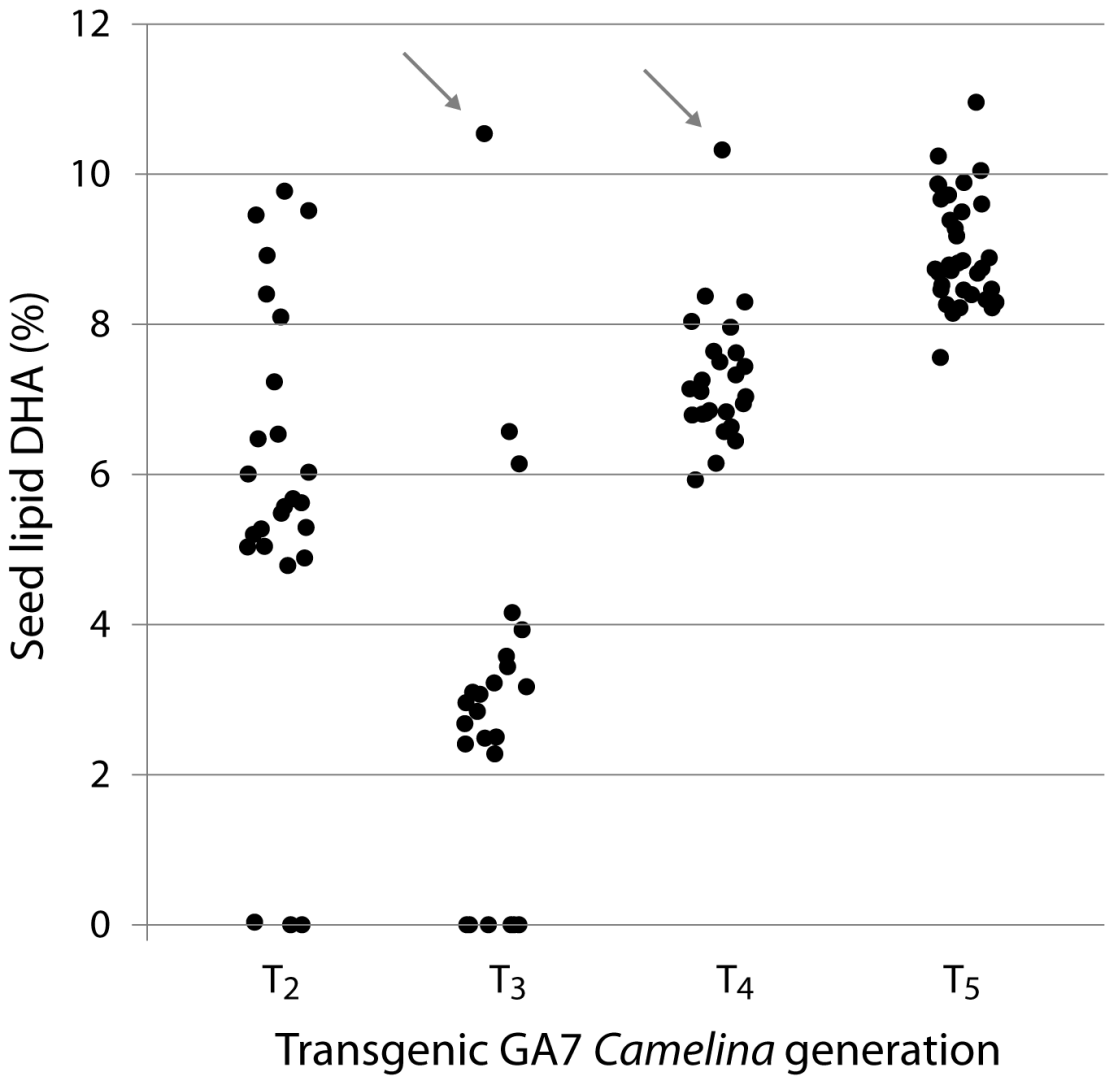
Relative level of DHA (as % of total fatty acids) in four generations of a transgenic multi-copy GA7 *Camelina sativa* line. The T_2_ generation shows single seed DHA levels, whilst pooled seed results (20 seeds per batch) are shown for T_3–5_ (T_4_ and T_5_ are not segregating). Arrows denote lines which were progressed to form the next generation.

**Figure 6:**
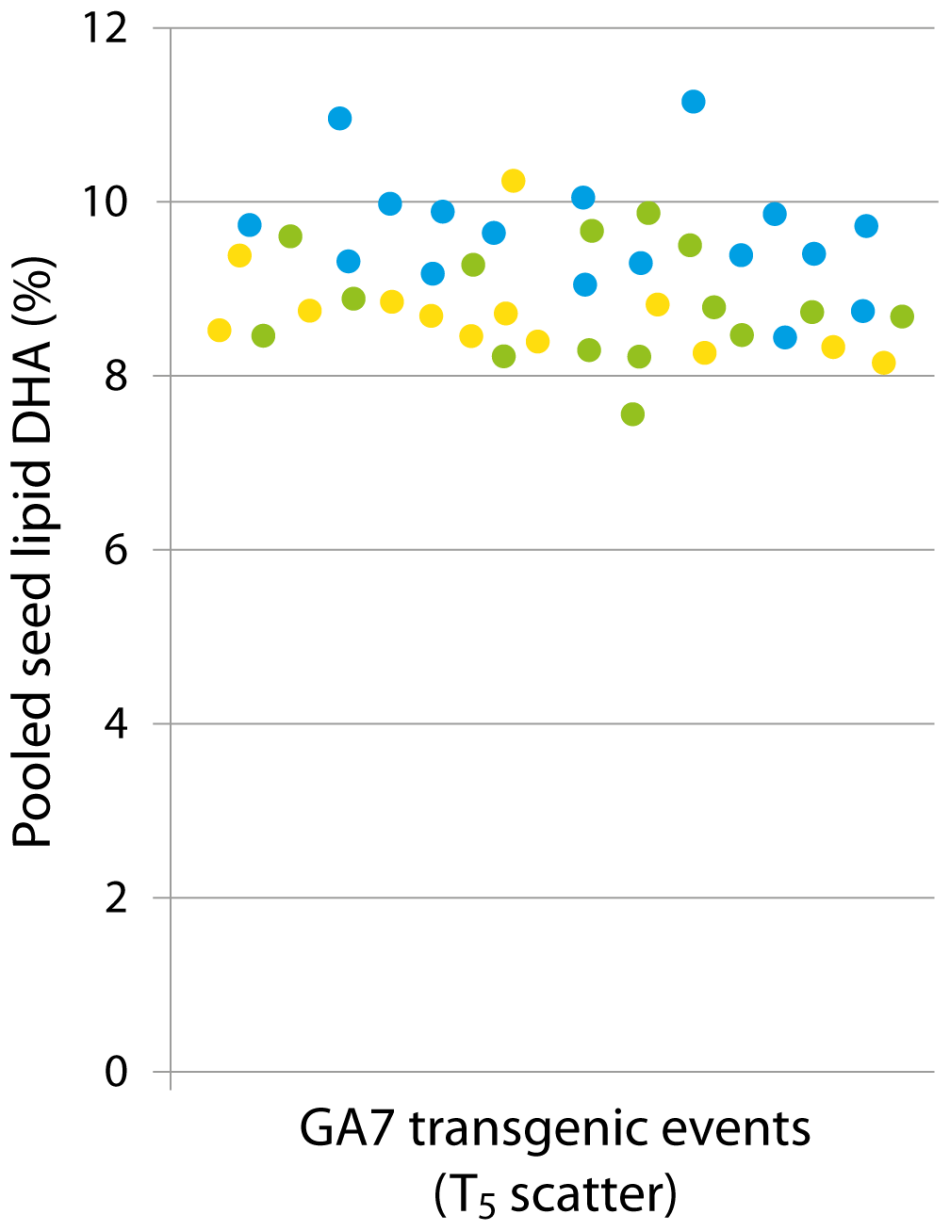
Relative level of DHA (as % of total fatty acids) of pooled seed from 60 randomly-selected plants from the T_5_ generation of GA7 transgenic. Events are colour-coded by glasshouse location (yellow, green and blue dots denote different glasshouses). The blue events were found to contain more DHA than the events in the other glasshouses (P<0.001).

**Figure 7:**
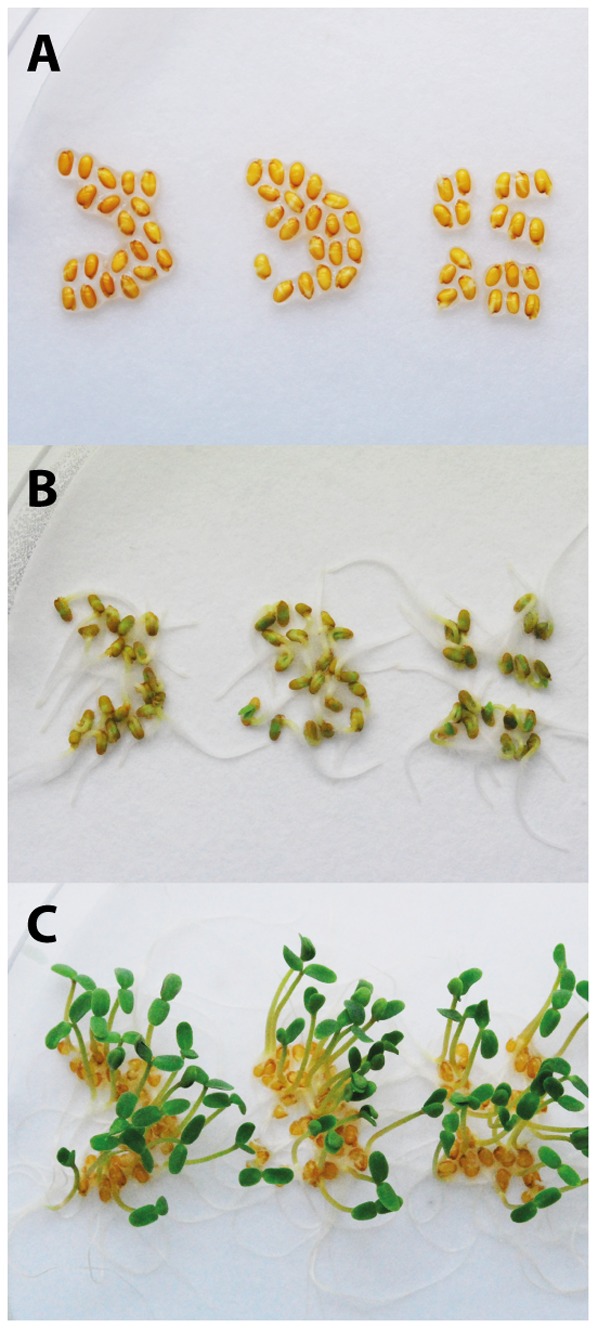
Germination of parental (left) and two transgenic GA7 *Camelina sativa* T_5_ lines (middle and right). A) 0 days following 3 days imbibition in the dark at 4°C; B) 1 day at 24°C under light; C) 4 days.

Seeds derived from the GA7 transgenic event with the highest DHA were sown out and subsequent generations established immediately to assess the trait stability over multiple generations. The maximum DHA levels observed was found to be stable to at least the fifth generation (Figure 5), although the pooled seed DHA level did not stabilise until T_4_ due to the presence of two transgenic loci. Interestingly, plants grown in one of the glasshouses contained significantly higher (P<0.001) levels of seed DHA than plants grown in other glasshouses (Figure 6). A more structured study is being performed to identify which environmental factors were responsible for this effect. T_5_ seed batches were also germinated on MS media alongside parental *C. sativa* seed with no obvious difference in germination rate or seedling vigour observed (Figure 7). The GA7 construct was transformed earlier than the modified versions and progressed through multi-generation characterisation rapidly. Similar multi-generation characterisation of mod-F and mod-G events is underway.

It is also important to note that the segregation ratios observed (∼3∶1 to ∼15∶1) indicate that one or, at most, two transgenic loci are required to produce fish oil-like levels of DHA in *C. sativa*. This has important implications for the ease with which the transgenic trait can be bred as well as for transgene stability. It was encouraging to observe that the GA7 DHA trait was stable to at least the fifth generation.

### EPA and DHA are located at *sn*-1/3 position in TAG

**Figure 8:**
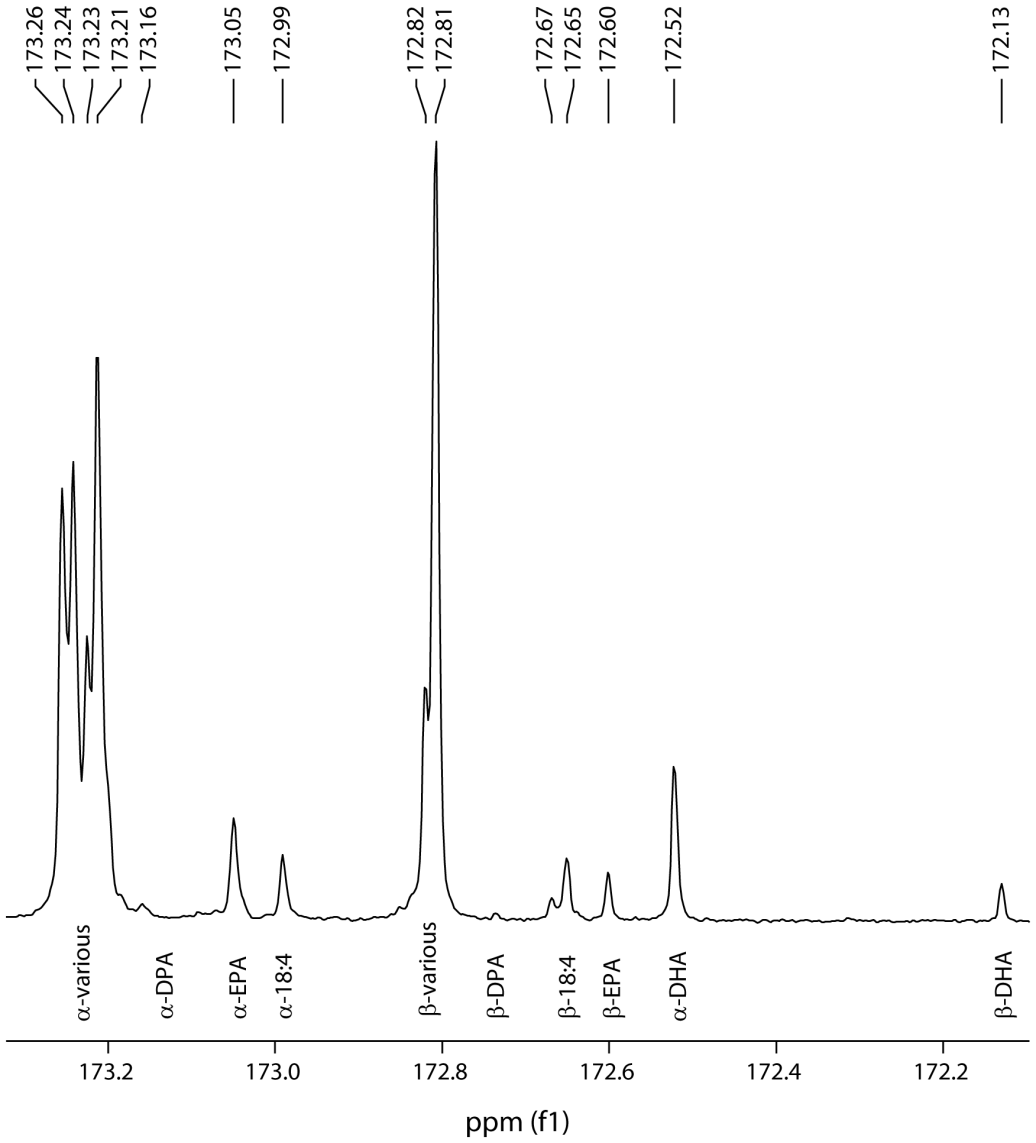
Trace of a ^13^C-NMR spectrum of the carbonyl (C1) region showing the positional distribution of EPA and DHA in transgenic *Camelina sativa* TAG with the *sn*-1,3 positions indicated by α and *sn*-2 by β. Both EPA and DHA are preferentially located at *sn*-1,3, although the effect is weaker for EPA.

^13^C NMR regiospecificity analysis was performed on the transgenic *C. sativa* seed oil to determine the positional distribution of the ω3 LC-PUFA on TAG (Figure 8). An event with approximately equal EPA and DHA was selected to maximise response for these fatty acids and the ratio of *sn*-1,3 to *sn*-2 was found to be 0.75∶0.25 for EPA and 0.86∶0.14 for DHA where an unbiased distribution would be 0.66∶0.33. This indicated that both fatty acids were preferentially located on the *sn*-1,3 positions in *C. sativa* TAG although the preference for EPA was weaker than for DHA. The finding that DHA was predominantly found on *sn*-1,3 was similar to results previously reported in *A. thaliana* seed [10] although the preferential location of EPA at the *sn*-1,3 position is in contrast with earlier studies which did not see such preference in linseed with EPA [4] or Arabidopsis with ARA, another C20 fatty acid [22]. It will be interesting to further identify positional distribution differences between host species.

## Conclusions

This study demonstrated the production of fish oil-like levels of DHA (12%) in transgenic *C. sativa* seed with low levels of intermediate fatty acid production and very high ω3: ω6 ratios with no new long-chain (≥C_20_) ω6 products. New ω3 fatty acids were found to accumulate in excess of 25% of total seed lipid. EPA and DHA were found to be enriched at the *sn*-1,3 positions in seed TAG although the effect was less strong for EPA. DHA was also found in the polar lipid fraction with the implication that the lecithin meal fraction would be similarly enriched for feed applications. The study also showed the importance of strong construct design when engineering complex multi-gene pathways in a single construct. DHA production by these constructs was found to be stable to at least the fifth transgenic generation.
