# Correction: Metabolic Engineering *Camelina sativa* with Fish Oil-Like Levels of DHA

**DOI:** 10.1371/journal.pone.0095409

**Published:** 2014-04-10

**Authors:** The PLOS ONE Staff

There is a typo in the first sentence of the abstract. Please read the correct sentence below:

Omega-3 long-chain (≥C20) polyunsaturated fatty acids (ω3 LC-PUFA) such as eicosapentaenoic acid (EPA) and docosahexaenoic acid (DHA) are critical for human health and development.
